# Genetic link between metabolic syndrome and coronary artery disease: Insights from genome-wide cross-trait analysis

**DOI:** 10.1016/j.gmg.2025.100092

**Published:** 2025-12-30

**Authors:** Pengcheng Yi, Quanting Yin, Huanhuan Zhang, Chunhua Yang, Yanping Zhu, Zhenhong Xia, Fuyi Xu, Jia Mi

**Affiliations:** a School of Pharmacy, Binzhou Medical University, Shandong, Yantai 264003, China; b Shandong Technology Innovation Center of Molecular Targeting and Intelligent Diagnosis and Treatment, Binzhou Medical University, Shandong, Yantai 264003, China

**Keywords:** Coronary artery disease, Diabetes, Obesity, Expression quantitative loci, Genetic correlation, Mendelian randomization, Co-localization

## Abstract

Metabolic syndromes (MeS), marked by central obesity, high blood pressure, abnormal cholesterol and blood sugar, are key cardiovascular disease (especially coronary artery disease, CAD) risk factors. Genetic studies show MeS-CAD genetic overlap, indicating shared biological pathways. We used Summary-data-based Mendelian Randomization (SMR), Bayesian colocalization (with large GWAS summary stats for MeS/CAD and cis-eQTL data from 3 tissues) and Transcriptome-Wide Association Study (TWAS). We also investigated the effects of gene knockout on mouse phenotypes. SMR found 886/737/192 shared genes in blood/brain cortex/liver; colocalization identified 11/13/5 shared causal genes in these tissues and 46 shared loci (e.g., CAMK1D, OR=1.11; AGPAT1, OR=1.13; FDR<0.05). Moreover, knocking out these genes in mice affected metabolism, adipose tissue, cardiovascular function, glucose homeostasis, and the fat/muscle balance. This study identified common regulatory genes between MeS and CAD, suggesting that targeted therapies or interventions could potentially address both conditions simultaneously, offering prospects for more integrated treatment strategies.

## Introduction

Coronary artery disease (CAD), characterized by the narrowing or blockage of coronary arteries that supply blood to the heart, represents a major cause of mortality worldwide, affecting both developed and developing nations [1], [2], [3]. The disease is strongly associated with several established risk factors, particularly metabolic syndrome (MeS) and its components, including type 2 diabetes (T2D), hypertension, hyperlipidemia, and obesity [1], [3], [4], [5]. Epidemiological evidence demonstrates that a 1 standard deviation increase in body mass index (approximately 5 kg/m²) correlates with a 10–20 % elevation in CAD risk [6], [7]. Furthermore, among T2D patients, a 1 % increase in baseline HbA1c levels has been shown to augment CAD risk by 2 % in African Americans and 6 % in European Americans [8].

MeS and CAD exhibit overlapping genetic variations, indicating potential shared biological pathways and therapeutic targets. MeS accelerates the development of atherosclerosis through various mechanisms, including hyperglycemia, dyslipidemia, and systemic inflammation, which significantly increase the risk of CAD [8], [9]. Genetic studies have revealed notable associations between MeS and CAD [1], [2], [4], [5], [10], highlighting polymorphisms in key genes. For example, two IL-6 gene polymorphisms have been linked to hypertension and CAD, providing insights into the role of inflammatory processes in the pathogenesis of both conditions [11]. Additionally, the FN1 (fibronectin 1) rs1250229-T polymorphism has been inversely associated with CAD in individuals with familial hypercholesterolemia, emphasizing the role of extracellular matrix remodeling in CAD progression [12]. Despite these findings, the underlying genetic mechanisms that connect MeS and CAD remain complex and not fully understood, Further research, particularly large-scale genomic studies, is needed to elucidate the precise genetic factors that contribute to the coexistence of MeS and CAD, which could pave the way for new strategies in prevention and treatment.

Genome-wide association studies (GWAS) have identified a substantial number of risk-associated genetic loci, laying the foundation for investigating the genetic basis of comorbidity inheritance [13], [14], [15]. Integrated analyses leveraging GWAS data, particularly independent and large-scale GWAS summary statistics, have emerged as powerful tools for identifying shared genes and biological pathways underlying comorbid conditions. Among these approaches, Summary-data-based Mendelian Randomization (SMR) and Bayesian colocalization have garnered significant attention in recent years due to their ability to efficiently analyze large-scale genetic datasets and uncover shared genetic mechanisms. SMR, an extension of traditional Mendelian Randomization, enables researchers to investigate causal relationships between genetic variants and complex traits by integrating GWAS data with xQTL data (including eQTL, pQTL, and mQTL) [14]. Bayesian colocalization, on the other hand, employs Bayesian statistics to estimate the posterior probability that a genetic locus is associated with multiple traits, thereby determining whether two or more traits share a common genetic basis [13]. These methodologies hold considerable promise for advancing our understanding of the genetic architecture underlying complex traits and diseases.

This study aims to elucidate shared biological pathways and therapeutic targets between MeS and CAD by integrating cis-eQTL data with GWAS summary statistics. We employed SMR and Bayesian colocalization to identify causal genes and shared genetic variants associated with both CAD and MeS. Through this approach, our study provides a theoretical framework for further exploration of the molecular mechanisms linking these conditions and offers valuable insights into potential genetic targets for therapeutic intervention.

## Materials and methods

### Data source

GWAS summary statistics for MeS were obtained from UKBiobank ICD PheWeb (https://pheweb.org/UKB-SAIGE/). The sample sizes for the traits were as follows: diabetes (20203 cases, 388756 controls), hyperlipidemia (35844 cases, 373034 controls), hypertension (77977 cases, 330366 controls), obesity (10799 cases, 397993 controls), T2D (18945 cases, 388756 controls). CAD GWAS summary statistics was downloaded from IEU OpenGWAS project (https://gwas.mrcieu.ac.uk/) with the accession ID: ebi-a-GCST005194 (34541 cases, 261984 controls).

Cis-eQTL summary statistics for liver (226 samples) and cortex (2865 samples) were downloaded from Yang Lab (https://yanglab.westlake.edu.cn/software/smr/#DataResource) [16], [17]. Circulating blood cis-eQTL data (31684 samples) was downloaded from eQTLGen (https://eqtlgen.org/cis-eqtls.html) [18]. All datasets were derived from European populations, and the reference genome used was GRCh37/hg19.

### SMR analysis

SMR was applied to investigate the causal associations between genetically determined endophenotype (blood, brain, and liver cis-eQTL) and complex traits of interest (MeS and CAD) [14], [19], [20], [21]. SMR analysis operates under the core assumption that gene expression (exposure) causally influences the phenotype (outcome). The analysis utilizes gene expression data from cis-eQTL summary statistics and phenotype data from GWAS summary statistics. The SMR analysis was performed using SMR software, version 1.3.1 (https://yanglab.westlake.edu.cn/software/smr/#Download). A False Discovery Rate (FDR) < 0.05 and HEIDI > 0.05 were set as the significance levels to filter out topSNPs. In the SMR analysis, we follow the standard methodology and use default parameters. The most significant cis-eQTL (P < 5e-8) within the cis-region of each gene probe was selected as the instrumental variable to assess the association between the gene's tissue-specific expression level and the outcome. We ensured allele frequency consistency across three datasets: GWAS summary statistics, eQTL summary data, and the LD reference panel (503 European individuals from the 1000 genomes project). SNPs showing allele frequency differences > 0.2 between any two datasets were excluded, with the total number of removed SNPs capped at 5 % of all variants. The HEIDI test excluded SNPs showing: (1) r² < 0.05 or > 0.9 with the top-SNP, and (2) pairwise r² > 0.9 among remaining SNPs (retaining only one per such pair). The numbers of cis-eQTL transcripts are 19,250 in blood, 16,744 in brain, and 4838 in liver.

### Bayesian colocalization analysis

To assess the likelihood that two traits share the same causal variant, we performed Bayesian colocalization analysis using the “coloc” package (version 5.2.3, https://github.com/chr1swallace/coloc) [13], [22]. The method provides five hypotheses: (1) no causal genetic variant for trait or tissue (PPH0); (2) one causal genetic variant in the region is associated with trait only (PPH1); (3) one causal genetic variant in the region is associated with tissue only (PPH2); (4) trait and tissue are associated with different genetic variants in the region (PPH3); (5) trait and tissue are associated with shared genetic variant in the region (PPH4) [23], [24], [25], [26]. In this study, we performed two types of Bayesian colocalization as follows. The differential thresholds for eQTL(PPH4 > 0.8) and phenotype-phenotype (PPH4 > 0.6) colocalization analyses were chosen based on their distinct objectives: eQTL colocalization required stringent thresholds (FDR < 0.05, HEIDI > 0.05 for SMR; PPH4 > 0.8) to prioritize variants with robust evidence of shared causal mechanisms between genes and phenotypes, minimizing false positives. Phenotype-phenotype colocalization adopted a more lenient threshold (PPH4 > 0.6) to capture broader associations, followed by validation through enrichment analysis and knockout models. This balanced rigor in causal inference with exploratory sensitivity.

#### eQTL colocalization

Single Nucleotide Polymorphisms (SNPs) within ±500 kb of top SNPs for blood, brain, and liver cis-eQTLs were selected (P < 5e-8) to perform colocalization analysis with MeS and CAD. Specifically, these top SNPs were derived from the results of our SMR analysis. PPH4 ≥ 0.8 was defined as strong evidence of shared causal variants [23], [24], [25], [26]. Minor Allele Frequency of liver cis-eQTLs were extracted from GTEx portal (https://www.gtexportal.org/home/downloads/adult-gtex/reference)^27^.

#### Phenotype-phenotype colocalization

SNPs within ±500 kb of top SNP from MeS and CAD GWAS summary statistics were selected for phenotype-phenotype colocalization. For each overlapping region (PPH4 > 0.6), gene annotations were downloaded from the Genome Data Viewer (https://www.ncbi.nlm.nih.gov/gdv/browser/).

### Transcriptome-wide association study (TWAS)

TWAS is a method used for exploring associations between gene expression and traits [28], [29], [30]. We conducted TWAS for diabetes, T2D, hypertension and childhood obesity (instead of obesity), using the TWAS Atlas database(https://ngdc.cncb.ac.cn/twas/downloads) [31]. Since there is no available data on obesity, we use childhood obesity data as a substitute. The analyses focused on five key tissues: adipose, whole blood, brain frontal cortex, brain cortex, and liver. The TWAS Atlas integrates high-quality gene-trait associations derived from multiple TWAS methodologies, including PrediXcan/S-PrediXcan, TWAS-FUSION, and UTMOST. Additionally, it incorporates results from complementary approaches such as SMR, transcriptome-wide Mendelian randomization (TWMR), kernel-based TWAS (kTWAS), and multi-omic strategies TWAS (MOSTWAS).

### Enrichment analysis

Enrichment analysis helps understand the biological processes and pathways by assessing the statistical enrichment of a given gene set [32], [33]. We performed a enrichment analysis on WebGestalt (https://www.webgestalt.org/) and assessed gene ontology in Biological Processes, pathway in KEGG, phenotype in Human Phenotype Ontology [34], [35], [36]. Significance level was set at FDR < 0.05.

### International mouse phenotyping consortium analysis

The International Mouse Phenotyping Consortium (IMPC, http://www.mousephenotype.org) aims to generate a functional directory of the mammalian genome by producing knockout mouse lines for every protein-coding gene, achieved through characterizing the phenotypes of mutants and controls [37]. IMPC provided the phenotype of mice harboring null alleles for Camk1d (Camk1d^em1(IMPC)Mbp^), Agpat1 (Agpat1^tm1.1(KOMP)Vlcg^), Ilrun (Ilrun^em1(IMPC)Tcp^), Tmem116 (Tmem116^tm1b(EUCOMM)Hmgu^) and Npepps (Npepps^em1(IMPC)Hmgu^). We specifically concentrated on examining the phenotypes related to growth, size, and body region, as well as those associated with homeostasis, metabolism, or adipose tissue, and also investigated the phenotypes pertaining to the cardiovascular system in these mice harboring null alleles.

## Results

### Overview of study design

**Fig. 1:**
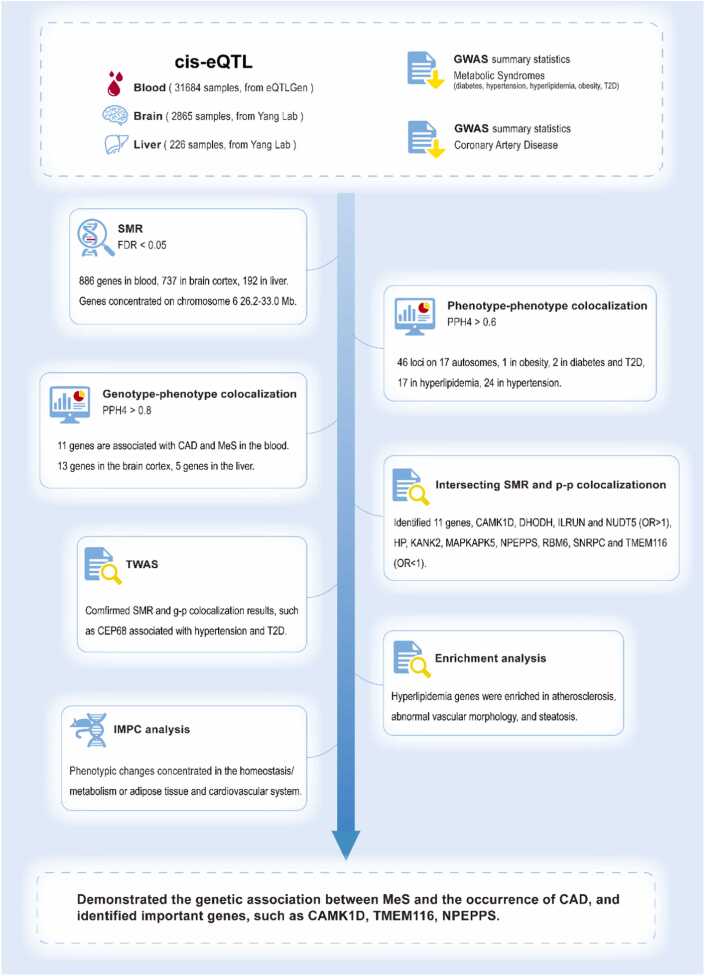
Overview of this study. Cis-eQTL and GWAS summary statistics data were used to conduct SMR analysis and identify the genes related to diseases. eQTL colocalization focused on genes that have passed SMR test (FDR < 0.05, HEIDI > 0.05). Intersecting SMR and phenotype-phenotype colocalization included the genes that have passed FDR but failed HEIDI. g-p,eQTL. p-p, phenotype-phenotype.

In this study, we systematically analyzed GWAS summary statistics for CAD and MeS, complemented by cis-eQTL data from blood, brain cortex, and liver, to identify shared genetic mechanisms. Our analytical framework employed: (1) SMR analysis (FDR < 0.05, HEIDI > 0.05) to detect tissue-specific causal genes; (2) Bayesian colocalization with differential thresholds - stringent (PPH4 > 0.8) for eQTLassociations to minimize false positives, and more inclusive (PPH4 > 0.6) for phenotype-phenotype relationships to capture weak but genuine phenotypic association signals; (3) TWAS and pathway enrichment for functional validation; and (4) IMPC mouse phenotyping to corroborate biological relevance (Fig. 1). By cross-validating findings from causal (SMR), variant-sharing (colocalization), and expression–trait (TWAS) association perspectives, and further validated through mouse phenotyping, these approaches jointly enhance the reliability of candidate genes.

### SMR analysis of MeS and CAD

**Fig. 2:**
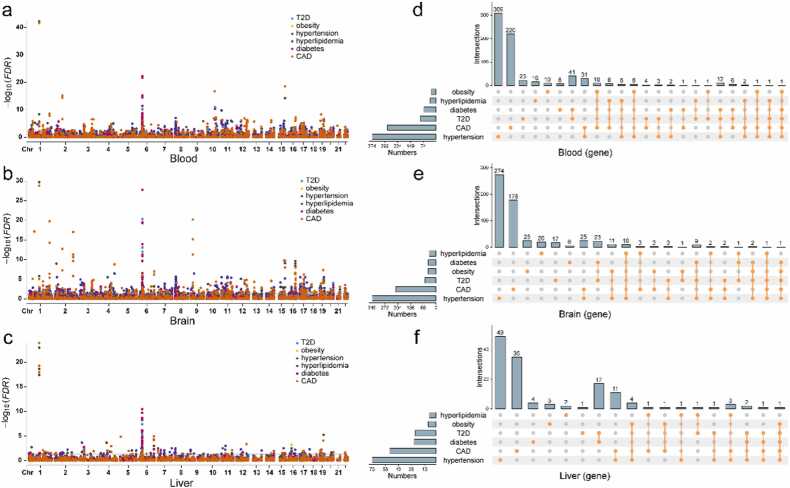
The SMR results of MeS and CAD. The Manhattan plot show the genes of SMR analysis of MeS and CAD in blood (a), brain (b) and liver (c). The genes of diseases were color-coded. The dash line indicates the significant threshold FDR = 0.05. d-f. The Upset plot show the connection of MeS and CAD in blood (d), brain (e) and liver (f). The mode of how to calculate the size of combination set is distinct.

To identify genes associated with both MeS and CAD from GWAS data, we conducted SMR analysis, revealing a total of 886 genes in blood (FDR < 0.05, HEIDI > 0.05; Fig. 2**a**), 737 in brain cortex (Fig. 2**b**), and 192 in liver (Fig. 2**c,** Table 1**,** Table S1) that demonstrated causal associations with MeS and CAD. The distribution of identified genes across the three tissues (blood, brain cortex, liver) for each disease was as follows: hypertension (374, 340, and 73), CAD (285, 215, and 53), T2D (93, 60, and 24), diabetes (71, 42, and 25), hyperlipidemia (34, 37, and 8), and obesity (29, 43, and 9).

**Table 1:** The genes associated with six diseases in three tissues were identified by SMR. The genes of each diseases have passed FDR and HEIDI test (FDR < 0.05, HEIDI > 0.05). The chromosome column only displays values > 10 %. Chromosome column: ‘X on Y (Z%)’ indicates X genes (out of the total disease-associated genes in the tissue) map to chromosome Y, accounting for Z% of the total.

**Diseases**	**Blood**		**Brain cortex**		**Liver**	
	Genes	Chromosome	Genes	Chromosome	Genes	Chromosome
CAD	285	29 on 17 (10.2 %)	215	27 on 17 (12.6 %)	53	-
Hypertension	374	41 on 17 (11.0 %)	340	-	73	8 on 16 (11.0 %)
Diabetes	71	9 on 6 (12.7 %)	42	-	25	14 on 6 (56.0 %)
Hyperlipidemia	34	4 on 6 (11.8 %)	37	6 on 6 (16.2 %)	8	2 on 6 (25.0 %)
T2D	93	20 on 6 (21.5 %)	60	9 on 6 (15.0 %)	24	14 on 6 (58.3 %)
Obesity	29	5 on 16 (17.2 %)	43	12 on 16 (27.9 %)	9	6 on 16 (66.7 %)
		10 on 17 (34.5 %)		10 on 17 (23.3 %)		2 on 17 (22.2 %)

Notably, genes associated with diabetes, hyperlipidemia, and T2D were predominantly clustered on chromosome 6 within the 26.2–33.0 Mb region, representing 56.0 % of the diabetes- and T2D-associated genes in the liver. This region included significant genes such as DDAH2 (OR *= 0.82*, FDR *= 8.46e-6*), which exhibited a pronounced effect on both CAD and MeS (excluding obesity). Furthermore, genes linked to obesity were primarily localized on chromosome 16 (28.4–31.3 Mb) and chromosome 17 (43.5–44.7 Mb) (Table 1**,** Table S1).

Among the identified genes, AIF1 (allograft inflammatory factor 1) in the brain cortex (OR > 1.10) and CKB (creatine kinase B) in the liver (OR < 0.95) were simultaneously associated with three MeS traits and CAD. AIF1, a calcium-binding protein implicated in macrophage activation, has been previously linked to the development of atherosclerosis [38]. CKB, a key regulator of energy transduction in tissues with high and fluctuating energy demands (such as skeletal muscle, heart, and brain), also plays a role in adaptive thermogenesis in brown adipose tissue. Notably, RBM6 (RNA-binding motif protein 6; OR < 1), which is known to inhibit cell proliferation, migration, and invasion, emerged as the only gene associated with four MeS traits (excluding hyperlipidemia) and CAD across all three tissues (Fig. 2d-f).

### eQTL colocalization

**Fig. 3:**
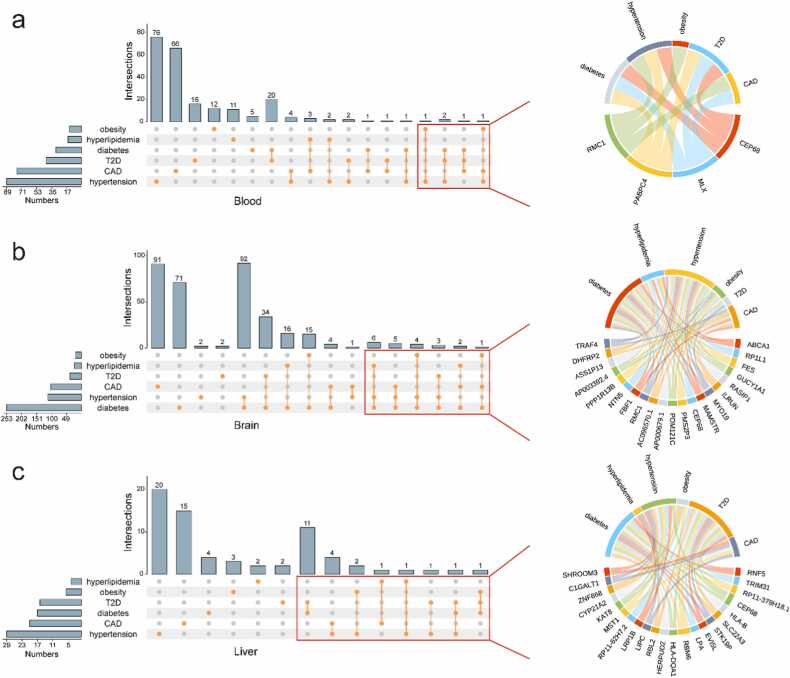
The correlation between diseases and genes in the eQTL colocalization. a-c. The Upset plot shows genes shared by six diseases in blood (a), brain (b) and liver (c). Genes were identified by Bayesian colocalization analysis. The significant level was PPH4 > 0.8. The chord plot shows the connection between six diseases and genes. Each gene in the chord plot is linked with more than two diseases.

To identify genes harboring shared causal variants between CAD and MeS, we conducted eQTL colocalization analysis. In blood, we identified 11 genes (*RN7SL600P*, MIR4513, EHMT2, PRKCE, RMC1, FES, LPIN3, *MRPL45P2*, PSMC3IP, MLX, *EIF2S2P3*) associated with both CAD and MeS (Fig. 3**a,** Table S2). Notably, elevated expression of MLX was linked to increased risks of CAD (OR=1.21), diabetes (OR=1.45), and T2D (OR=1.45). In the brain cortex, 13 genes (ABCA1, DHFRP2, AC096887.2, AP003392.4, TDRKH-AS1, TRAF4, TDRKH, PPP1R13B, ILRUN, AP000679.1, FBF1, LDLR, AIF1) demonstrated associations with both CAD and MeS (Fig. 3**b,** Table S2). In the liver, 5 genes (MST1, C1GALT1, SHROOM3, CYP21A2, LIPC) were found to be associated with both conditions (Fig. 3**c,** Table S2). Among these, CYP21A2 expression showed a negative correlation with the risk of CAD (OR=0.97) and hypertension (OR=0.96).

Building upon these findings, we further validated candidate genes through a multi-stage analytical approach. First, we performed SMR analysis to identify genes with causal effects on phenotypes (FDR < 0.05, HEIDI > 0.05). The intersection of SMR results with TWAS findings from the TWAS Atlas database yielded 13 validated genes (DDAH2, NME6, SPINK8, UNC13D, ZDHHC18, FBF1, UBE2Z, C1GALT1, CYP21A2, NOTCH4, MYO19, RBM6, SLC22A3). Among these, NOTCH4 (P value = 9.65e-17), DDAH2 (P value = 5.93e-16), and CYP21A2 (P value = 1.02e-8) demonstrated particularly significant associations with various diseases. eQTL colocalization analysis revealed that a subset of these genes, including CYP21A2, RBM6 and SLC22A3, showed strong evidence of shared causal variants (PPH4 > 0.8).

**Fig. 4:**
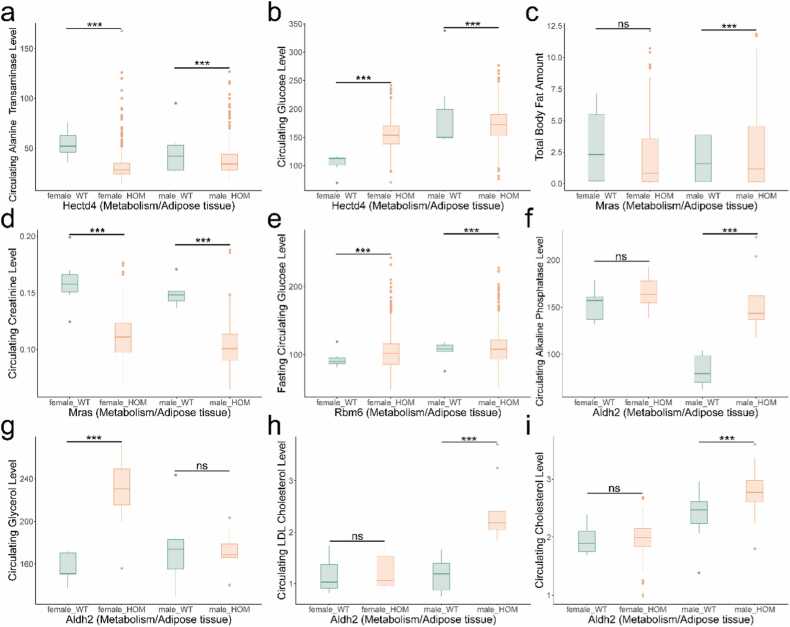
The role of eQTL colocalization genes in different knockout mice. Hectd4 (a-b), Mras (c-d), Rbm6 (e) and Aldh2 (f-i) knockout mice exhibited phenotype changes in metabolism/ adipose tissue. Life stage is early adult. HOM, homozygous. ns, P value > 0.05. *, P value < 0.05. **, P value < 0.01. ***, P value < 0.001.

To assess the functional relevance of these genes, we examined knockout mouse models from the International Mouse Phenotyping Consortium (IMPC). This analysis revealed four genes with notable metabolic phenotypes (Fig. 4). Hectd4 knockout mice exhibited decreased circulating alanine transaminase levels and increased circulating glucose levels, with distinct gender-specific effects (Fig. 4**a-b**). Mras knockout mice showed reduced circulating creatinine levels (Fig. 4**c-d**). Rbm6 knockout mice displayed decreased fasting circulating glucose levels (Fig. 4**e**). The Aldh2 knockout model demonstrated significant gender-specific phenotypic differences: male mice exhibited increased circulating alkaline phosphatase, LDL cholesterol, and total cholesterol levels, while female mice showed elevated circulating glycerol levels (Fig. 4**f-i**).

### phenotype-phenotype colocalization

**Fig. 5:**
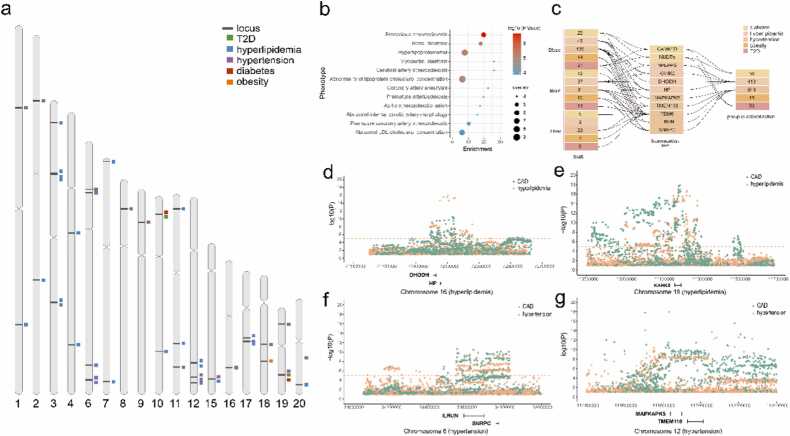
Loci and genes of phenotype-phenotype colocalization. a. The distribution of loci which shared causal variant with MeS and CAD in chromosome. b. Phenotype enrichment analysis for phenotype-phenotype colocalization genes (P value < 1e-4). c. The intersection genes of the SMR analysis and phenotype-phenotype colocalization for MeS and CAD. The genes of SMR and phenotype-phenotype colocalization column are the intersect results of MeS and CAD. phe-phe, phenotype-phenotype. d. The plot showing the relative position of genes and SNPs. The dash line indicates significant threshold P value = 1e-5.

To further investigate shared genetic architecture, we performed phenotype-phenotype colocalization analysis, identifying 46 shared loci between MeS and CAD (PPH4 > 0.6) distributed across 17 autosomes (Fig. 5**a**). The distribution of loci and corresponding genes identified by SMR for each MeS trait was as follows: obesity (1 locus, 18 genes), diabetes (2 loci, 36 genes), type 2 diabetes (T2D, 2 loci, 36 genes), hyperlipidemia (17 loci, 413 genes), and hypertension (24 loci, 549 genes). Enrichment analysis revealed that the associated phenotypes were primarily concentrated in atherosclerosis and lipid metabolism pathways (P < 1e-4, Fig. 5**b**). Notably, several overlapping genes—including PCSK9, APOC2, APOC3, APOA1, APOA5, ABCG5, ABCG8, LDLR, LPL, and PCK1—have been previously implicated in both lipid metabolism and cardiovascular disease.

In total, we identified 11 common genes that were consistently detected in both the SMR analysis (excluding HEIDI results) and phenotype-phenotype colocalization analysis (Fig. 5**c,** Table S3). Specifically, elevated expression of CAMK1D, DHODH, ILRUN, and NUDT5 was associated with an increased risk for both MeS and CAD (OR > 1). In contrast, higher expression of NPEPPS, RBM6, SNRPC, and TMEM116 demonstrated protective effects against MeS and CAD (OR < 1). Notably, HP, KANK2, and MAPKAPK5, which are linked to hypertension, hyperlipidemia, and CAD, exhibited tissue-specific effects, with risk-increasing associations in blood (OR > 1) but protective effects in the brain (OR < 1). Additionally, several genes within these loci, such as KANK2 and TMEM116, passed the SMR analysis (Fig. 5**c**).

**Fig. 6:**
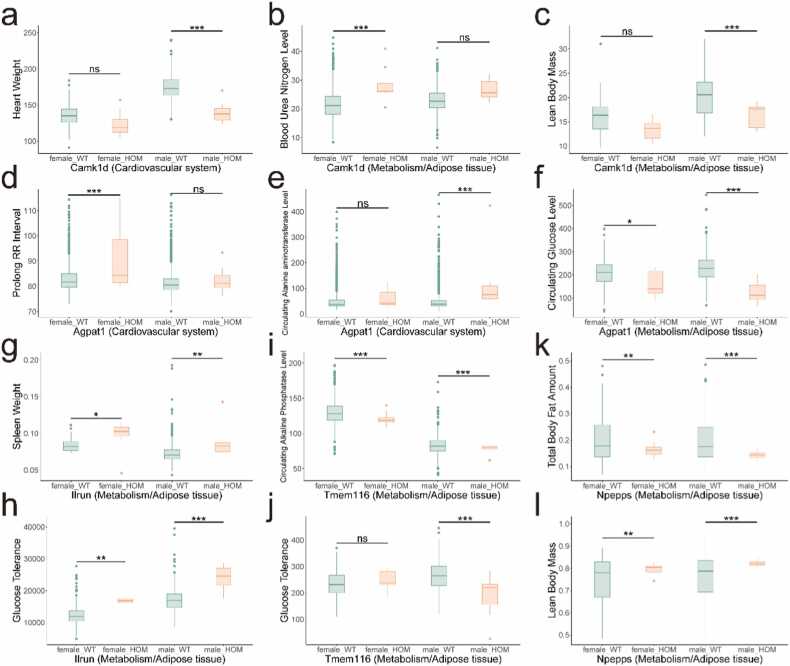
The role of phenotype-phenotype colocalization genes in different knockout mice. The phenotype changes of Camk1d (a-c) and Agpat1 (d-f) knockout mice included cardiovascular system and metabolism/adipose tissue. Ilrun (g-h), Tmem116 (i-j) and Npepps (k-l) knockout mice exhibited phenotype changes in metabolism/adipose tissue. d. The interval between electrocardiograms was prolonged. Life stage is early adult. Glucose tolerance, Intraperitoneal glucose tolerance test (IPGTT), the charts show the results of measuring Area under glucose response curve. HOM, homozygous.

We further explored phenotypic alterations in several knockout mouse models. Six knockout models (Camk1d, Npepps, Hpse, Tmem116, Rbm6, Ilrun) exhibited significant changes in metabolism and homeostasis, while one model (Camk1d) additionally demonstrated alterations in the cardiovascular system (Fig. 6). Specifically, Camk1d knockout male mice displayed reduced lean body mass and decreased heart weight, whereas female mice showed elevated blood urea nitrogen levels (Fig. 6**a-c**). Agpat1 knockout mice exhibited prolonged electrocardiogram intervals, suggesting a potential role in cardiac function (Fig. 6**d-f**). Ilrun, previously implicated in glucose metabolism, was associated with changes in spleen morphology and weight (Fig. 6**g-h**). Tmem116 knockout mice demonstrated decreased circulating alkaline phosphatase levels and impaired glucose tolerance in males (Fig. 6**i-j**). Additionally, Npepps knockout resulted in increased lean body mass and reduced total body fat, indicating its involvement in fat metabolism and muscle production (Fig. 6**k-l**).

## Discussion

The relationship between metabolic diseases and CAD is complex and potentially bidirectional, suggesting an underlying genetic regulatory basis [6], [11], [39], [40]. For instance, increased adiposity has been causally linked to a higher risk of CAD, supporting obesity as a significant risk factor for CAD [6], [11], [12]. Our research has further elucidated this relationship by identifying key genes (such as DDAH2 and CYP21A2) that regulate both MeS and CAD. These findings contribute to the growing evidence of shared genetic mechanisms between MeS and CAD, while nominating candidate genes for further investigation as potential therapeutic targets. These findings could inform future research toward personalized approaches, pending replication in independent cohorts and functional validation.

We identified a significant clustering of causal genes within specific chromosomal regions. Genes associated with diabetes, hyperlipidemia, and type 2 diabetes (T2D) were predominantly concentrated in the 26.2–33.0 Mb region on chromosome 6. Previous genome-wide significant meta-analysis (GSMA) studies on T2D, obesity, and CAD have revealed shared genetic loci among these three diseases, particularly within the 6q23.2 - q25.3 region on chromosome 6 and the 16q12.2-q23.1 region on chromosome 16 [41]. Notably, the imprinted locus at 6q24 on chromosome 6 has been established as the primary genetic cause of transient neonatal diabetes, a condition that often recurs after puberty [42]. Our findings deepen the understanding of the complex relationship between diseases and specific chromosomal loci, offering valuable insights for the development of targeted therapeutic strategies in the future.

The genes we identified on chromosome 6 are associated with protective effects against MeS and CAD. Notably, several of these genes are located within the major histocompatibility complex (MHC) region—a genomic locus characterized by extraordinary genetic diversity, dense gene clustering, and intricate regulatory mechanisms that govern immune responses and multiple complex traits. Despite the analytical challenges posed by the MHC region’s high linkage disequilibrium (LD) and functional complexity, the genes we identified here have well-established associations with MeS and CAD, supporting the robustness of our findings. Among these (DDAH2, HLA-DQA1, CYP21A2), DDAH2, a protein-coding gene encoding Dimethylarginine Dimethylaminohydrolase 2 (OR *= 0.82*), plays a critical role in catalyzing the metabolic conversion of ADMA into citrulline [43]. ADMA, an endogenous inhibitor of nitric oxide synthase, has been linked to cardiovascular diseases when present at elevated concentrations [44]. Notably, the genetic polymorphism (−499 C/G rs805305) within the DDAH2 gene is significantly associated with plasma ADMA levels in CAD patients, although it may not directly confer CAD risk in the Chinese population [43], [45]. Furthermore, DDAH2 demonstrates protective effects against diabetes, hypertension, and type 2 diabetes. Additionally, DDAH2 has been shown to mitigate myocardial fibrosis in diabetic cardiomyopathy [46]. In our study, DDAH2 demonstrated significant associations with reduced risk of MeS and CAD in blood-based analyses, suggesting its potential role as a candidate therapeutic target and biomarker. When combined with ADMA levels, this association may offer insights for CAD risk stratification, though further validation is needed to assess clinical utility.

By integrating SMR and Bayesian colocalization analyses, we identified several key genes with significant implications. CAMK1D (Calcium/Calmodulin Dependent Protein Kinase 1D), a protein-coding gene involved in transferase activity (transferring phosphorus-containing groups) and protein tyrosine kinase activity, has been recognized as a risk factor for CAD and represents a potential druggable target [47]. Notably, male mice with Camk1d gene knockout exhibit ghrelin resistance, resulting in reduced weight gain and protection against high-fat diet-induced obesity [48]. Our findings demonstrate an association between CAMK1D (OR=1.04) and an elevated risk of CAD. Additionally, Camk1d knockout in male mice leads to decreased heart weight and lean body mass. We further hypothesize that CAMK1D may protect the cardiovascular system against fibrosis, hypertrophy, and apoptosis by regulating NOS3, GUCY1A2, GUCY1A1, and GUCY1B1, thereby reducing the incidence of cardiovascular diseases. Together with CYP21A2, CAMK1D is implicated in cholesterol-related pathways through our analyses, positioning it as a plausible candidate gene linking cardiovascular and metabolic traits.

TMEM116 (Transmembrane Protein 116) contains SNPs associated with non-synonymous amino acid changes, and its expression levels are correlated with CAD loci across multiple tissues, demonstrating a protective effect against CAD (OR=0.93). Furthermore, knockout of the TMEM116 gene in male mice leads to decreased circulating alkaline phosphatase levels and impaired glucose tolerance [49]. Multiple bioinformatics analyses have confirmed a close relationship between CAD and T2D, mediated by the central gene NPEPPS, which is involved in cell metabolism and plays a significant role in phosphatidylinositol metabolism. This suggests that NPEPPS may have critical implications in the progression of T2D [50]. Notably, knockout of the NPEPPS gene in mice results in reduced total body fat mass and increased lean body mass.

In summary, this study enhances our understanding of the genetic regulatory mechanisms linking MeS to CAD. The identified genes provide a robust foundation for further exploration of shared molecular mechanisms and biological processes underlying MeS and CAD. Future research focusing on biomarkers and cell-type-specific analyses of these conditions will enable more accurate risk assessment for specific populations and contribute to improved diagnostic and therapeutic strategies for CAD.

## Author contributions

FX and JM conceived the study. PY conducted and performed data analysis. PY wrote the manuscript. QY and HZ prepared the figures. YZ, ZX, CY, FX, and JM reviewed the manuscript. All authors read and approved the final version of the manuscript for publication.

## Ethics approval and consent to participate

All data used in this study were obtained from public databases and do not involve any ethical concerns. Clinical trial number: not applicable.

## Consent for publication

All authors have agreed to publish.

## Funding

This research was funded by Taishan Scholars Construction Engineering, 10.13039/501100001809National Natural Science Foundation of China (32170989), Major Basic Research Project of Shandong Provincial Natural Science Foundation (ZR2019ZD27), Key Research and Development Program of Shandong Province (2023CXPTO12), 10.13039/501100007129Natural Science Foundation of Shandong Province (ZR2021MH141, ZR2023MH373), Binzhou Medical University Research Start-up (50012305190).

## Declaration of Competing Interest

The authors declare no competing interests.

## Data Availability

All the data involved in this study were obtained from public databases. This study code is available on GitHub (https://github.com/yi9099/genetic-link), and is free for academic and research purposes.

## Appendix A Supplementary material

Table S1, the results of SMR analysis. Table S2, the results of eQTL colocalization. Table S2, the results of phenotype-phenotype colocalization

## Footnotes

Appendix A Supplementary data associated with this article can be found in the online version at doi:10.1016/j.gmg.2025.100092.
